# A Multi-Center Observation Study on Medication-Related Osteonecrosis of the Jaw (MRONJ) in Patients with Osteoporosis, and Other Non-Malignant Bone Diseases, in Northwestern Italy over 16 Years

**DOI:** 10.3390/biomedicines12102179

**Published:** 2024-09-25

**Authors:** Dora Karimi, Paolo Giacomo Arduino, Alessio Gambino, Francesco Erovigni, Alessandro Dell’Acqua, Francesco Pera, Massimo Carossa, Monica Pentenero, Paolo Appendino, Francesco Della Ferrera, Antonella Fasciolo, Majlinda Caka, Mario Migliario, Matteo Brucoli, Stefano Franchi, Alessandro Pezzimenti, Vittorio Fusco

**Affiliations:** 1 Department of Surgical Sciences, Cir-Dental School, University of Turin, 10126 Turin, Italy; dora9k@hotmail.it (D.K.); alessio.gambino@unito.it (A.G.); ferovigni@cittadellasalute.to.it (F.E.); adellacqua@cittadellasalute.to.it (A.D.); francesco.pera@unito.it (F.P.); massimo.carossa@unito.it (M.C.); 2 Oral Medicine and Oral Oncology Unit, Department of Oncology, San Luigi Gonzaga Hospital, University of Turin, 10043 Turin, Italy; monica.pentenero@unito.it; 3 Department of Dentistry and Oral Surgery, Mauriziano Hospital, 10128 Turin, Italy; pappendino@mauriziano.it (P.A.); frankiedef86@gmail.com (F.D.F.); 4 Maxillofacial Surgery Unit, Azienda Ospedaliera Universitaria “SS Antonio e Biagio e C. Arrigo”, 15121 Alessandria, Italy; afasciolo@ospedale.al.it; 5 Maxillofacial Surgery Unit, Asti Hospital, 14100 Asti, Italy; mcaka@asl.at.it; 6 Dental Clinic, Department of Health Science, University of Eastern Piedmont “A. Avogadro”, 28100 Novara, Italy; mario.migliario@med.uniupo.it; 7 Maxillofacial Surgery, Novara Hospital, 28100 Novara, Italy; matteo.brucoli@med.uniupo.it (M.B.); stefano.franchi@ospfe.it (S.F.); 8 Independent Researcher, 10100 Turin, Italy; sandro.p85@libero.it; 9 Oncology Unit, DAIRI Department, Azienda Ospedaliera Universitaria “SS Antonio e Biagio e C. Arrigo”, 15121 Alessandria, Italy; fusco.dott.vittorio@gmail.com

**Keywords:** osteonecrosis of the jaw, osteoporosis, bisphosphonates, alendronate, denosumab, incidence

## Abstract

Objectives: To assess the number of new cases of Medication-Related Osteonecrosis of the Jaw (MRONJ) among patients with osteoporosis and other non-malignant bone diseases in Northwestern Italy between 2007 and 2022. Methods: MRONJ cases were collected from referral centers in a population of 4.5 million. We analysed the number of new MRONJ cases per year, type of disease, administered drugs, duration of therapy (when available), and onset time of disease. Results: We analysed 198 cases (out of 1071 total MRONJ cases); diseases included osteoporosis (87%), rheumatoid arthritis (5%), their association (4%), Paget’s disease, and other various diseases (4%). Patients received bisphosphonates alone (74%), or denosumab alone (6%), or a sequence of different drugs (20%). The number of new cases increased over five years from 2 (2003–2007) to 51 (2008–2012), 65 (2013–2017), and 79 (2018–2022), and the percentage increased from 1% to 14%, 20%, and 29% of the total cases. Conclusions: The number of new MRONJ cases per year among patients with non-malignant diseases is rapidly increasing all around the world, though underestimation cannot be excluded. In this study, we describe epidemiological and clinical characteristics of patients, and the drug most frequently involved in MRONJ cases in our region over a long period, allowing a comprehensive view of the progression of the disease. Greater collaboration among specialists is needed for correct and early diagnosis to improve measures potentially reducing disease incidence and to limit quality of life deterioration in patients with osteoporosis and other non-malignant diseases.

## 1. Introduction

Osteonecrosis of the jaw (ONJ) in patients receiving antiresorptive drugs (bisphosphonates and denosumab) or other drugs is now referred to as MRONJ (Medication-Related Osteonecrosis of the jaw) [1]. MRONJ has been recognized for its potential impact on the quality of life of both cancer and myeloma patients, as well as those with osteoporosis and other non-malignant diseases [2]. A widely adopted definition based on an 8-week observation of bone exposure in the oral cavity was partially expanded in 2014 [1,2,3], but it remains questionable, due to cases without bone exposure or fistula, and the underestimation of imaging’s role in diagnosis [4].

The real incidence and prevalence of MRONJ in the general population is still unknown. Epidemiology data are scarce due to problematic coding of the disease and inadequacies in data from adverse reaction surveillance systems [5,6]. Insurance databases or large national healthcare system databases, even those using specific algorithms for data extraction, have shown significant limitations or inconsistent results [7,8,9,10,11,12,13]. Collection of clinically confirmed MRONJ cases outside of clinical trial settings is difficult [14], and few studies have attempted to report clinical data from patient charts in a specified population [15,16,17,18,19,20]. A large Scandinavian registry of all clinically confirmed MRONJ cases observed in three nations has been established [14,21], and results are awaited. The risk of MRONJ is higher in patients with bone metastases from various cancers and in myeloma patients receiving high doses of Bone Modifying Agents (BMAs), including bisphosphonates (e.g., zoledronic acid and pamidronate infused intravenously monthly or quarterly; oral or intravenous ibandronate at high doses) and monthly subcutaneous injections of denosumab 120 mg [4,22]. The MRONJ risk is reported to be generally much lower in patients with osteoporosis and other non-malignant diseases receiving low doses of BMAs, including oral bisphosphonates (alendronate, risedronate), delayed doses of zoledronic acid (5 mg annually), low dose ibandronate (oral monthly 150 mg or intravenous 3 mg quarterly), or denosumab (60 mg every six months) [3,6,22].

An attempt to ascertain epidemiological data in Northwestern Italy was conducted, collecting a relatively high number of MRONJ cases in cancer and myeloma patients by examining the clinical charts of a public cancer network [19,20]. Collaterally, oral care centers collaborating with the network collected MRONJ cases among patients with osteoporosis and other non-malignant diseases to evaluate the frequency of MRONJ in this latter patient population. Here, we report available data for MRONJ cases evaluated by oral care centers over 16 years in patients with non-malignant diseases. To the best of our knowledge, this is the biggest case series of such patients from Italy.

## 2. Materials and Methods

Since November 2005, the regional ONJ Study Group and Supportive Care Study Group, established by members of the Piedmont and Valle d’Aosta cancer network (Rete Oncologica) in Northwestern Italy, collected data on MRONJ cases observed at each center of the network [19,20]. All referral oral care centers (Maxillofacial Surgery, Oral Medicine and Oral Surgery units) in the region collaterally collected MRONJ cases outside of the main studied population (patients with cancer and bone metastases or myeloma) to evaluate possible differences in clinical features and to expand experience in therapeutic management of MRONJ. A collection of cases among patients with non-malignant diseases was conducted after cross-checking reports from oral care centers to avoid double counting. Only forms with sufficient data were included in an appropriate database.

Data collection concerning disease history and occurrence of MRONJ was further refined by two authors (D.K., A.G.) who visited all the involved units (*n* = 11). MRONJ cases included those with bone exposure or fistula and no history of radiation therapy according to the definition of the American Association of Oral and Maxillofacial Surgeons (AAOMS) [3], as well as cases without clear bone exposure or fistula, according to Italian (SIPMO-SICMF) clinical and radiological criteria [6].

The following data were registered and analysed: (a) demographics: age, sex; (b) type of disease: osteoporosis (including osteopenia), rheumatoid arthritis, other bone or autoimmune diseases; (c) antiresorptive therapy: agent (first reported therapy and any second drug administered in sequence), date of therapy start, and duration of treatment (when available); (d) site of involvement reported as maxillary vs. mandible or both; (e) year of MRONJ diagnosis.

Median (interquartile range, IQR) or mean (standard deviation, SD) were applied to continuous data, while numbers (percentage) were used for categorical data. Analyses were carried out using STATA SE version 15.1 (STATA Corp, College Station, TX, USA).

## 3. Results

Between January 2007 and December 2022, we have collected data on 1071 MRONJ cases. Of these, 198 developed osteonecrosis due to antiresorptive treatment for bone diseases (osteoporosis, osteopenia, Paget’s disease, etc.), other than metastatic cancer or myeloma.

### 3.1. Patients’ Characteristics

The main characteristics of the 198 MRONJ cases in patients without bone metastatic cancer or myeloma are presented in Table 1. Females comprised 97% of the entire sample; the mean age at diagnosis time was 75 years (SD +/− 9.91) and median age was 76 years (range 37–98). Of 198 patients, year of first MRONJ diagnosis was reported in all cases (see Table 2 and Table 3). Osteoporosis alone was the most commonly reported disease (173 cases; 87%), followed by Rheumatoid Arthritis (9 cases; 5%), osteoporosis together with Rheumatoid Arthritis (8 cases; 4%), and other types of diseases, such as Paget’s disease (with 2 cases), lupus, giant cell arthritis, erosive arthritis, undifferentiated connective tissue disease, femoral coxitis and polymyalgia rheumatica (8 cases; 4%).

### 3.2. Drug and Duration of Therapy

Overall, the administered antiresorptive drug (bisphosphonates, denosumab) as first or only agent was alendronate (118; 59.6%), ibandronate (34; 17.2%), zoledronic acid (10; 5.1%), risedronate (13; 6.6%), denosumab (12; 6.1%), clodronate (6; 3%), neridronate (3; 1.5%), and pamidronate (2; 1%). Out of 198, 158 patients (80%) underwent treatment with one antiresorptive alone, while the remaining 40 (20%) were administered a sequence of antiresorptive agents, with denosumab (22 cases) being the most frequent second drug. Denosumab 60 mg every six months was administered as first drug in 12 cases and in sequence (before or after other bisphosphonate) with other drugs in 23 cases: alendronate (11 patients), ibandronate (6), zoledronic acid (4), clodronate (1), risedronate (1). In patients treated with only one drug, the reported duration of therapy was available for only 114 patients; this ranged between 1 and 30 years (mean 6.8 years; median 5 years), with 46 patients between 1 and 3 years, 43 between 4 and 9 years, and 25 patients with 10 or more years of treatment. (Table 3). The second drug was administered for 1–9 years (mean 2.8 years, median 2 years) at the time of MRONJ diagnosis. The overall duration (first and second drug) ranged between 1 and 30 years.

### 3.3. Clinical Manifestation

Most patients (135; 68.2%) had a necrotic lesion in the mandible; in 50 patients (25.2%) MRONJ lesions occurred in the maxilla, whereas in 13 patients (6.6%) it occurred in both maxilla and mandible. Multiple sites of MRONJ were reported in 34 cases (17%).

### 3.4. Number of ONJ Cases by Period of Data Collection

**Figure 1:**
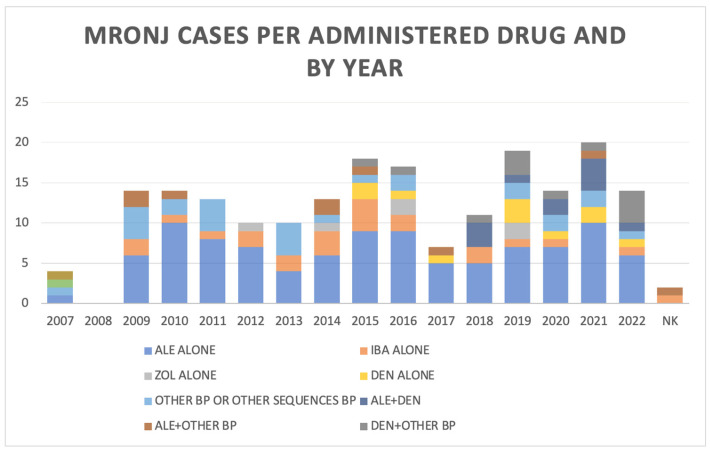
Number of MRONJ cases per year of diagnosis (global and per drug or sequence). ALE = alendronate; BP = bisphosphonate; DEN = denosumab; IBA = ibandronate; ZOL = zoledronic acid; NK = not known.

Figure 1 shows the distribution of MRONJ diagnosis per year, according to the main agent/sequence of drugs administered. Being 198 cases reported along 16 years, we observed a mean of 12.4 cases per year, with a median of 13 (range 0–21).

### 3.5. Survival Rate

According to latest evaluation (December 2023) of the data of the regional registry office service, 26 patients (13%) were dead and 173 (87%) still alive.

## 4. Discussion

To date, proper incidence and prevalence of MRONJ in patients exposed to antiresorptive drugs, due to osteoporosis and other non-malignant diseases, are still unknown. As well as for cancer and myeloma patients, accurate studies are not easily interpretable in the literature, due to several issues, including (but not limited to) heterogeneity and challenging accountability of the exposed population (patients with osteoporosis versus rheumatologic or autoimmune diseases); uncertainty of diagnosis and adjudication of MRONJ disease; variability of clinical data collection of suspected and ascertained cases; eventual changing of the time of the therapy in osteoporosis and autoimmune patients; and discrepancy between sources of data (for example reporting to Drug Safety Pharmacovigilance agencies versus clinical surveys and multi-center studies). Consequently, large case MRONJ collections in defined populations (e.g., regional, or national) are needed and could be useful to evaluate the real frequency of the disease.

The available published data for epidemiology of ONJ have been obtained from case series, retrospective observational studies, and cohort studies, such as insurance claims data. Lo and coworkers [7] provided a reliable example of the incidence of ONJ. They surveyed 13,946 patients who had received oral BP therapy in the Kaiser Permanent of Northern California database and found possible ONJ cases. Then, these possible ONJ cases were confirmed according to the American Association of Oral and Maxillofacial Surgeons (AAOMS) criteria and incidence was estimated at 28 per 100,000 person-years. Tennis and coworkers [22] showed that, in an osteoporosis cohort, the incident was 15 per 100,000 person/years among those who used oral bisphosphonates. It is typically difficult to directly compare the results among different regions or states, because there is no specific ICD code for ONJ and no verified claim-based algorithms, so incidence rates vary depending on the algorithm identifying possible ONJ cases across studies [9,10,23]. Only a registry of all the cases observed in a defined population could potentially give more refined epidemiology information [14,15,16,17,18,19,20,21]. In most worldwide experiences registered in oral and maxillofacial surgery centers, the absolute number of MRONJ cases among cancer and myeloma patients (receiving high doses of bisphosphonates and/or denosumab) is generally higher than that among patients with osteoporosis and non-malignant diseases (treated with low doses of BMAs) [6]. However, even if the individual MRONJ risk is much lower, the large number of patients exposed to BMAs due to osteoporosis justifies an expected rise in the number of MRONJ cases among non-malignant patients.

In previous research, our regional study group within the Piedmont and Valle d’Aosta network (Rete Oncologica) collected data on frequency of MRONJ among cancer patients from 2009 to 2018 [19,20]. It showed a steady incidence of MRONJ per year from 2009 to 2015 via the retrospective data collection, with a mean of 51.3 cases per year, and a raw unadjusted incidence of 11.6 MRONJ cases/million/year. Conversely, during the 2016–2018 period, when data were collected prospectively from the six main collaborating oral care centers, they observed a mean of 33.3 cases per year, with a raw unadjusted incidence of 7.5 MRONJ cases/million/year [20]. On the other hand, in this study among patients with non-malignant diseases throughout the 16-year period of investigation, we found an increasing trend emerging, with a median of 11.5 (range 0–14) yearly MRONJ cases in the 2007–2014 period, which increased to 15.1 (range 7–21) yearly MRONJ events in the 2015–2022 period. We expected a similar trend in the following years, based on the hypothesis that MRONJ occurrence would be influenced by ageing of population and drug accumulation effect. Among patients with MRONJ developed after “low dose” antiresorptive drugs in our regional experience, most patients received bisphosphonates (principally oral bisphosphonates, such as alendronate, residronate, or oral ibandronate). Low-dose denosumab therapy has been introduced in Italy for non-metastatic patients since 2013, either administered as a first-line treatment against bone metabolic disorders, or as a “switch” drug from protracted bisphosphonate treatment. Consequently, cases of MRONJ after denosumab therapy have been registered since 2015, with 11 cases from patients undergoing treatment with denosumab alone, and 23 cases in patients switching from another bisphosphonate to denosumab (or vice versa). In a population of about 4.5 million people, we registered 79 cases in 2018–2022 among patients with osteoporosis and non-malignant diseases, a number higher than in previous periods. We can infer a raw incidence of 3.5 MRONJ cases/million/year in 2018–2022 related to low dose BMAs in our region. This result is inferior to that deducible by Swedish data in the Skane region: in fact, Hallmer and coworkers [16] registered 31 ONJ cases in osteoporosis patients in 4 years (2012–2015) in a population of 1.3 million, for an inferred raw incidence of 5.9 cases/million/year, even higher than that among cancer patients in the same region. In a recent outstanding Japanese research project, in the prefecture of Hyōgo (about 5.5 million people) [24], 1021 MRONJ cases were registered in three years (550 among patients receiving low dose BMAs and 471 among cancer/myeloma patients receiving high dose BMAs), with apparent incidence much higher than previously reported.

Regarding the median latency between the start of therapy and onset of MRONJ (Table 3), we found significant difference among the main therapy groups. Most MRONJ cases are observed starting from the fifth year of treatment, except for patients treated with “low dose” zoledronic acid (median 2.5 years) and denosumab 60 mg (median 2.6 years). However, the wide range observed among all the treatment groups implies that each single patient might already become at risk of MRONJ after a few months of therapy, as well as after many years, with cases of MRONJ diagnosed after 30 years (of treatment or follow-up) since the start of antiresorptive treatment. The main strength of the present work relies on the long-term duration of the observation and data collection, in a restricted territory (Piedmont and Valle d’Aosta) with a relatively well-defined population (now 4.5 million inhabitants), which implies the interception of most (if not all) cases occurring in real life practice.

One limit of the study relies on the heterogeneous approach required for the collection of the data, with data from 2009 to 2015 acquired retrospectively, whilst a prospective effort has been carried out since mid-2015. In any case, data collection for the second period was based on cases from the six main and larger oral care centers (which had observed 98% of the cases registered in the first period). We cannot exclude that less severe MRONJ cases might not have been addressed by dental practitioners on referral to oral care centers. Further observation time is certainly necessary. Another limit of the current and previous studies is the impossibility to obtain the exact incidence and prevalence of MRONJ in a population of non-metastatic patients in our territory, due to the lack of data about the precise number of patients undergoing each one of the aforementioned treatments (low dose BMAs: bisphosphonates, denosumab) in Piedmont-Valle d’Aosta.

In summary, the present series aligns with the findings of our previous paper [19] and recent literature [6,24,25,26]: MRONJ cannot be considered as a rare adverse event and should require continuous attention and awareness by prescribers of high dose BMAs (oncologists and haematologists) as well as of low dose BMAs (practitioners, rheumatologists, and other bone specialists), oral and maxillofacial specialists, dental practitioners, and health policy stakeholders. Our study demonstrates an increasing trend in MRONJ cases among patients with non-malignant diseases over a 16-year period. This trend highlights the need for heightened awareness and more collaborative efforts for early diagnosis and prevention among healthcare providers managing osteoporosis and other non-malignant bone diseases.

## 5. Conclusions

Due to the large number of studies, MRONJ is well-known in patients with malignant diseases treated with high dose anti-resorptive drugs. On the other hand, the difficult in obtaining epidemiological data for patients with non-malignant bone diseases probably underestimates the real number of new MRONJ cases in this specific group of patients. In this study we describe epidemiological and clinical characteristics of patients, and the drug most frequently involved in MRONJ cases in our region, with the advantage of a long period of study.

Despite the existence of papers with a long period of follow-up [27], our task had the unique peculiarity of describing specifically non-malignant diseases with 16 years of follow-up, allowing a comprehensive view of the progression of the disease over the years in a restricted territory with a well-defined population. In our experience, MRONJ appeared to be increasing in frequency over the last 16 years among patients affected by bone metabolic disorders, occurring so far with predilection for mandible sites, in elderly (>70 years) and female patients, mostly during/after bisphosphonate(s) treatment. This could be due to a better awareness of the related problems in dentists and other medical categories. MRONJ cases solely related to denosumab (60 mg every 6 months) are so far anecdotal, although such frequency might arise in upcoming years, due to an increasing propensity for prescription of denosumab as first-line treatment against bone metabolic disorders, as well as a “switch” drug from protracted bisphosphonate regimens. Further recollection of clinical data—concerning comorbidities, oral health risk factors, differential radiological features, and surgical outcomes—should be pursued, to clarify the main characteristics of these novel clusters of patients, emerging out of the wide population of osteoporotic elders undergoing low-dose bisphosphonates and low-dose denosumab.

However, considering the increasing number of patients with MRONJ, further studies to develop strategic treatment and preventive measures are recommendable. The study underscores the critical need for greater collaboration among medical and dental specialists for early diagnosis and improved management strategies of MRONJ in patients with non-malignant bone diseases. Early intervention and preventive measures are essential to reduce the incidence and mitigate the impact on patients’ quality of life.

## Figures and Tables

**Table 1:** Main characteristics of MRONJ patients.

	N	%
Total	198	
Age	Mean: 75 years (SD 9.91 years)	
Gender		
Female	192	97
Male	6	3
Bone disease		
Osteoporosis	173	87
Rheumatoid arthritis	9	5
Osteoporosis and rheumatoid arthritis	8	4
Other osteo-metabolic disease *	8	4
Antiresorptive treatment		
Alendronate only	100	50.5
Ibandronate only	23	11.6
Zoledronic acid only Denosumab only	6 11	3 5.6
Sequence ALE/DEN or vice versa	11	5.6
Sequence ALE/oBP or vice versa Sequence DEN/oBP or vice versa	9 12	4.5 6.1
Other BPs or other sequences	26	13.1

* Paget’s disease (2), lupus (1), giant cell arthritis (1), erosive arthritis (1), undifferentiated connective tissue disease (1), femoral coxitis (1), polymyalgia rheumatica (1). BP = bisphosphonate; oBP = other bisphosphonate (s); ALE = alendronate DEN = denosumab.

**Table 2:** MRONJ cases, categorized for drug administered and year of diagnosis.

DRUG/YEAR	2007	2008	2009	2010	2011	2012	2013	2014	2015	2016	2017	2018	2019	2020	2021	2022	NK	TOT
ALENDRONATE	1	0	6	10	8	7	4	6	9	9	5	5	7	7	10	6	0	100
ZOLEDRONATE	0	0	0	0	0	1	0	1	0	2	0	0	2	0	0	0	0	6
DENOSUMAB	0	0	0	0	0	0	0	0	1	1	1	0	3	1	2	1	0	11
CLODRONATE	0	0	0	0	0	0	0	0	0	0	0	0	1	1	1	1	0	4
IBANDRONATE	0	0	2	1	1	2	2	3	4	2	0	2	1	1	0	1	1	22
NERIDRONATE	0	0	0	0	1	0	0	0	0	0	0	0	0	0	1	0	0	2
PAMIDRONATE	0	0	1	0	0	0	0	0	0	1	0	0	0	0	0	0	0	2
RISEDRONATE	1	0	0	2	1	0	3	1	1	0	0	0	1	0	0	0	0	10
TOT	2	0	9	13	11	10	9	11	16	15	6	7	15	10	14	9	1	158
ALE + DEN			0	0	0	0	0	0	0	0	0	3	1	2	4	1	0	11
ALE + oBP			2	1	0	0	0	2	1	0	1	0	0	0	1	0	1	9
ZOL + DEN			0	0	0	0	0	0	1	0	0	0	1	0	0	2	0	4
ZOL + oBP			1	0	1	0	1	0	0	0	0	0	0	1	0	0	0	4
DEN + oBP			0	0	0	0	0	0	0	1	0	1	2	1	1	2	0	8
oBP sequence			2	0	0	0	0	0	0	1	0	0	0	0	0	0	0	4
TOT			5	1	1	0	1	2	2	2	1	4	4	4	6	5	1	40

ALE = alendronate; oBP = other bisphosphonate (s); DEN = denosumab; ZOL = zoledronic acid; NK = not known.

**Table 3:** Duration of antiresorptive treatment at the MRONJ onset time.

DRUG(S)	No.	Median Duration and Range (Year)	25% Interquartile	75% Interquartile
ALENDRONATE	100	8.2 (1–30)	3.7	11.1
ZOLEDRONIC ACID	6	2.5 (0–7)	1.0	3.9
DENOSUMAB	11	2.6 (1–4)	2.1	3.0
IBANDRONATE	22	4.8 (2–8)	3.0	7.5
OTHER BP ALONE	19	5 (0–15)	1.4	5.7
ALE + DEN OR VICE VERSA	11	8.1 (3–10)	7.9	9.9
ALE + IBAN OR VICE VERSA	5	5 (3.1–6.2)	3.8	5.4
ALE + oBP or VICE VERSA	4	7 (3–10)	6.2	9.7
DEN + oBP OR VICE VERSA	12	7 (3–19)	3.5	9
OTHER BP SEQUENCES	8	6 (0.5–14)	2.8	8.1

ALE = alendronate; IBAN = ibandronate; oBP = other bisphosphonate (s); DEN = denosumab.

## Data Availability

Data are not publicly available, due to privacy restrictions; however, authors (D.K. and A.G.) can provide details upon direct specific request.
